# Optimizing photoexcitation conditions for time‐resolved X‐ray solution scattering experiments

**DOI:** 10.1002/2211-5463.70304

**Published:** 2026-07-15

**Authors:** Matteo Levantino

**Affiliations:** ^1^ ESRF – The European Synchrotron Grenoble France

**Keywords:** laser photoexcitation, multiphoton absorption, protein dynamics, signal‐to‐noise ratio, time‐resolved X‐ray solution scattering

## Abstract

Investigating the dynamics of biological molecules in real time is crucial to understand their biological function. Time‐resolved X‐ray solution scattering (TR‐XSS) is an experimental technique that allows one to follow large‐scale conformational changes of biological molecules in solution induced by a triggering event. In many cases, the triggering event is the absorption of a laser pulse, either by the biological molecule of interest or by photocaged compounds present in solution. Selecting the optimal photoexcitation conditions is a critical step in a TR‐XSS experiment and it requires taking into account the laser beam properties, the sample characteristics, and the photoexcitation geometry. This protocol describes how to select the accessible experimental parameters in order to maximize the TR‐XSS signal while avoiding nonlinear effects induced by an excessive laser peak power density. The provided set of guidelines will aid the experimentalists in the preparation and realization of their TR‐XSS experiments.

## Graphical abstract

Time‐resolved X‐ray solution scattering (TR‐XSS) is a powerful technique to visualize how proteins change their structure in real time after light activation. Selecting the right laser photoexcitation conditions—fluence, excitation geometry, and sample refresh rate—is critical to maximize the experimental signal while avoiding unwanted side effects. This protocol provides practical guidelines to help researchers optimize their TR‐XSS experiments.

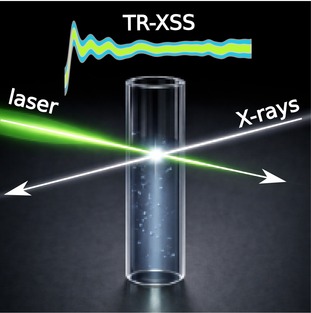

**Abbreviations**
AdoCbl: adenosylcobalamin
ESRF: European Synchrotron Radiation Facility
FWHM: full width at half maximum
NIR: near‐infrared
OCP: orange carotenoid protein
PDB: Protein Data Bank
RMS: root‐mean‐square
SNR: signal‐to‐noise ratio
TR‐SFX: time‐resolved serial femtosecond crystallography
TR‐XSS: time‐resolved X‐ray solution scattering
*Tt*CarH: *Thermus thermophilus* CarH

In an attempt to educate users in the field of time‐resolved femtosecond X‐ray protein crystallography (TR‐SFX), Schlichting and coworkers and Dwayne‐Miller and coworkers published a series of articles indicating that the number of absorbed photons per molecule has to be 1 [1, 2, 3, 4]. Although this rule‐of‐thumb recommendation is appropriate in the specific case of highly concentrated (≈ 10 mm) protein crystals exposed to femtosecond‐long laser pulses, this should not be extrapolated to other more general contexts. The most insidious phenomenon that can happen in the case of high laser peak power density is multiphoton absorption. By definition, multiphoton absorption is a nonlinear process in which a chromophore undergoes an electronic transition from a lower to a higher energy state with the simultaneous absorption of two or more photons. Multiphoton absorption can potentially lead the chromophore to explore many different (depending on how many photons are absorbed simultaneously) high‐energy states that are normally never populated. The probability of these nonlinear processes decrease, however rapidly with the laser peak power density: the photons need to be close enough in space and in time to be absorbed by the same chromophore in a single process. Multiphoton absorption is different from the sequential absorption of several photons by the same chromophore: if the time in between two sequential absorption processes is long enough, the effect of each photon absorption becomes indistinguishable from that of the following one. In an intermediate case, although the chromophore may not have enough time to fully relax to the ground electronic state, it will in general have undergone some (vibrational) relaxation to lower energy states. Clearly, also in this case, one needs to be cautious about potential induced modifications of macromolecular function (see eg [5, 6]). Nevertheless, the probability of such phenomena is in many cases negligible, provided that the peak power density is on the order of ≈ 0 $\mathrm{GW}\cdot cm^{-2}$ or lower [4], even in cases where more than one photon is absorbed by each chromophore within the duration of a single photoexcitation laser pulse. It should be also noted that, in the case of high peak power density laser beams, other molecules different than the target chromophore might undergo transitions due to multiphoton absorption, thus further increasing the total energy absorbed by the system and the probability of triggering side reactions.

The above considerations by no means aim at suggesting that laser photoexcitation conditions should not be kept under control. On the contrary, in preparing any time‐resolved experiment and a TR‐XSS one in particular, it is critical to estimate all relevant parameters that contribute to ensure an efficient photoexcitation of the system under investigation, while minimizing undesired side effects. In the following, we show how parameters like the sample absorbance, laser fluence, laser peak power, number of absorbed photons per chromophore, and the sample flowing speed can be calculated and tuned with reference to real experimental cases. This protocol is mainly aimed at young researchers who are already familiar with the TR‐XSS method but are interested in profiting from a more quantitative approach to their experiments, allowing more efficient data collections in well‐defined photoexcitation conditions. General information about the TR‐XSS method is available in recent literature reviews [7, 8, 9, 10]. More detailed information on data reduction and analysis of TR‐XSS data is reported in a different protocol published in the present issue by Andersson and coworkers [11].

## Materials

### TR‐XSS datasets

The experimental TR‐XSS datasets reported in the current protocol are publicly available in the ESRF DOI Portal (DOIs: 10.15151/ESRF‐ES‐704502367 and 10.15151/ESRF‐ES‐1437867878). Details on the sample preparation and on any experimental details not explicitly stated in this protocol can be found in Rios‐Santacruz *et al*. [12].

### TR‐XSS setup

TR‐XSS data were collected at the ID09 beamline of the European Synchrotron Radiation Facility (ESRF). ID09 is a beamline dedicated to time‐resolved X‐ray experiments with time‐resolution down to 100 ps [13]. Experiments on biological molecules are typically performed using a nanosecond laser tunable from the near ultraviolet to the near infrared. The polychromatic X‐ray pulses generated from an in‐vacuum undulator (magnetic period of 17 mm, fundamental energy tunable in the 14.7 to 19 keV range) are chopped to a maximum repetition rate of 1 kHz by means of a system of mechanical choppers [14]. A high‐speed chopper allows extracting either single 100 ps X‐ray pulses or a microsecond‐long train of pulses. The maximum attainable flux at a sample position in the case of 100 ps pulse selection mode is $10^{9}$ photons/pulse. A gain in flux of up to a factor of 1000 can be obtained by selecting a microsecond‐long train of pulses (the train pulse duration can be varied between 5 and 100 μs). The sample is typically flown by means of a flow‐through capillary with a thin (10 μm) glass wall connected to either a peristaltic or a syringe pump, so that each pump‐pulse pair hits a fresh portion of the sample.

### Software

Datasets were analyzed with custom‐made Python scripts based on the publicly available *pytxs* package (https://pypi.org/project/pytxs/).

## Methods

Below are described a series of steps that are required in a typical TR‐XSS scattering experiment. The focus is on the investigation of large conformational changes of biological systems that can be directly triggered through photoexcitation of a specific chromophore or indirectly by photoexcitation of a photocaged compound.

### Absorption spectrum

**Fig. 1:**
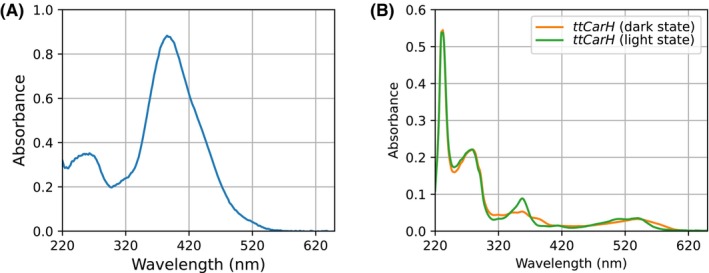
Optical absorption spectra of (A) Acid Yellow 9 (blue curve), a dye used to estimate solvent heating contributions in TR‐XSS (time‐resolved X‐ray solution scattering) data and (B) *Thermus thermophilus* CarH (*Tt*CarH) in the dark state (orange curve) and its light‐activated state (green curve). The spectra were collected using a Nanodrop One spectrophotometer (Thermo Fisher Scientific, Waltham, MA, USA) with the “automated pathlength” option activated. Absorbance values (A) are defined according to Eq. 1, where $A=\log_{10}\left(I_{0}/I\right)$; $I_{0}$ is the incident light intensity and I is the transmitted light intensity.

One of the first steps in the preparation of a TR‐XSS experiment is to collect an optical absorption spectrum of a sample having composition and chromophore concentration as close as possible to those that will be employed in the TR‐XSS experiment. The goal is to obtain an accurate estimation of the sample absorbance at the wavelength that will be used for laser photoexcitation. As an example, Fig. 1 reports the spectrum of Acid Yellow 9 and that of *Thermus thermophilus* CarH (*Tt*CarH), both in its dark state and its light‐activated state. Acid Yellow 9, also called Fast Yellow, is a dye commonly used for estimating the solvent heating contribution in TR‐XSS data. *Tt*CarH is a protein photoreceptor recently investigated with TR‐XSS [12], which contains a vitamin $B_{12}$ derivative called adenosylcobalamin (AdoCbl).

The sample absorbance is defined as:

(1) $$A=\log_{10}\left(\frac{I_{0}}{I}\right)$$

where $I_{0}$ is the incident light beam intensity and I is the transmitted light beam intensity. As for any optical absorption measurement where the detector shot noise is the main source of uncertainty, it is important to ensure that the absorbance is in the 0.5 to 1.5 range (at least at the photoexcitation wavelength):

(2) 0.5≤A≤1.5

Significant deviation from the above range will result in higher uncertainties on the measured absorbance. Note that, in the case of commercial spectrophotometers using microliter droplets of sample, the light pathlength is typically adjusted automatically by modifying the thickness of the liquid column in between two optical fibers. In these cases, data are reported in absorbance equivalent as if the sample thickness was 1 cm. Extra caution should be taken in these cases, as a peak absorbance of 1.0 in 1 cm equivalent units might correspond to an actual peak absorbance outside the range defined by Eq. 2. For example, in the case of the data reported in Fig. 1, while the Acid Yellow 9 spectrum was measured using a pathlength of 0.1 mm and resulted in a peak absorbance of 0.88, the *Tt*CarH spectra were measured with a 0.2 mm pathlength and have an absorbance lower than 0.1 in the AdoCbl absorption band at ~520 nm. In this last case, the spectrophotometer automatically selected the 0.2 mm pathlength, as the next available pathlength (1 mm) will have resulted in an absorbance higher than 1.5 at the 230 nm peak. Another important point to consider is that of buffer absorption, especially when the photoexcitation wavelength is in the UV. In the following, we assume that buffer absorption is negligible at the laser photoexcitation wavelength. However, it is important to check that this assumption is correct by measuring the absorption spectrum of the buffer solution independently.

### Signal‐to‐noise

**Fig. 2:**
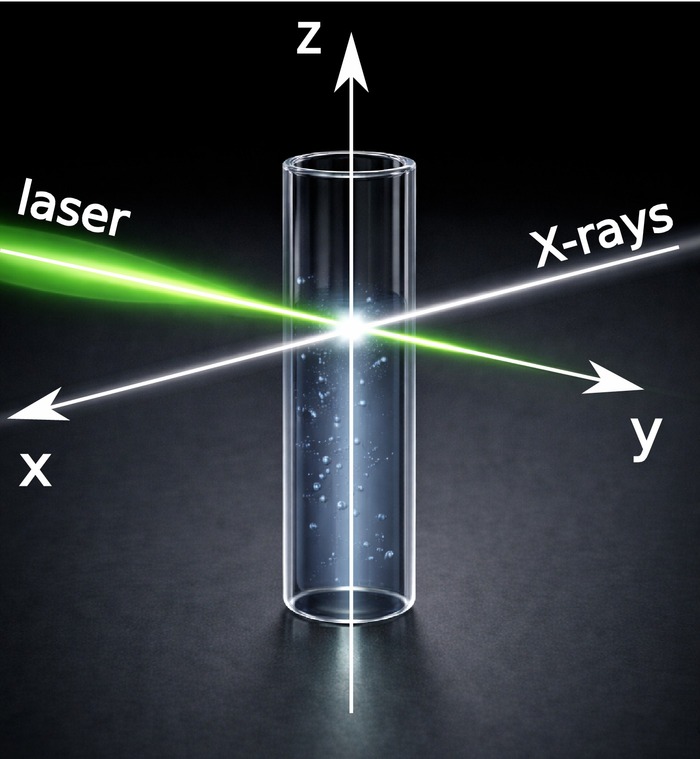
Typical geometry of a TR‐XSS (time‐resolved X‐ray solution scattering) experiment. The sample flows through a glass capillary. The laser beam (green) propagates along y and is focused at the capillary. The X‐ray beam (light gray) propagates along x and intercepts the sample in the laser photoexcited sample volume.

Once the absorbance spectrum of the sample has been determined, it might be necessary to dilute/concentrate the sample in order to have an absorbance at the photoexcitation wavelength that will maximize the signal‐to‐noise ratio (SNR) of the TR‐XSS signal. This optimal absorbance depends, however, on the photoexcitation geometry. In the following, we consider two cases: collinear and orthogonal excitation between the X‐ray and the laser beam. In both cases, we assume that the sample is contained (or flowing through) a glass capillary and that the X‐ray and laser beam are in a plane perpendicular to the capillary axis. Figure 2 depicts the case of orthogonal geometry.

As shown in the Supporting Information (cf. Eqs. S13 and S17), the signal is proportional to the difference between the scattering from a photoexcited protein, $s_{\text{prot}}^{\text{light}}$, and the scattering from a protein in its “dark” state (the protein equilibrium state in the absence of photoexcitation), $s_{\text{prot}}^{\text{dark}}$:

(3) $$\begin{array}{rcl} \Delta S\left(qt\right) & \;=\; & S_{\text{light}}\left(q,\;t\right)\;-\;S_{\text{dark}}\left(q\right)\\ & = & N_{\text{prot}}^{\text{light}}\;\left[s_{\text{prot}}^{\text{light}}\left(q,\;t\right)\;-\;s_{\text{prot}}^{\text{dark}}\left(q\right)\right] \end{array}$$

where $S_{\text{light}}\left(q,t\right)$ is the scattering pattern of the sample at time t from photoexcitation, $S_{\text{dark}}\left(q\right)$ is the scattering pattern of the sample before photoexcitation, and $N_{\text{prot}}^{\text{light}}$ is the number of photoexcited proteins in the X‐ray probed volume.

The associated noise is instead proportional to the square root of the total scattering, $S\left(q\right)$. As shown in the Supporting Information (cf. Eq. S21), the SNR can be written as:

(4) $$\mathrm{SNR}\;=\;\frac{\;N_{\text{prot}}^{\text{light}}\;\;\left[s_{\text{prot}}^{\text{light}}\left(q,\;t\right)\;-\;s_{\text{prot}}^{\text{dark}}\left(q\right)\right]\;}{\sqrt{2\;S\left(q\right)}}$$

It is important to stress that the total scattering $S\left(q\right)$ in Eq. 4 contains several contributions: not only the scattering from protein molecules, but also the scattering from the solvent (buffer) and the background scattering (air scattering, capillary scattering, etc.). All these contributions are essential in determining the SNR associated with the TR‐XSS signal, especially at wide scattering angles, where the SNR is typically lower than in the Guinier region.

#### Collinear geometry

The number of proteins undergoing a structural change after photoexcitation ($N_{\text{prot}}^{\text{light}}$) is proportional to the number of absorbed photons $N_{\mathrm{abs}}$:

(5) $$N_{\text{prot}}^{\text{light}}\;=\;\kappa_{\text{light}}\;N_{\mathrm{abs}}$$

where $\kappa_{\text{light}}$ is the photoexcitation yield, ie the fraction of proteins that are undergoing a structural change out of all of the proteins that have absorbed a photon.

In the case of collinear geometry, $N_{\mathrm{abs}}$ is equal to:

(6) $$N_{\mathrm{abs}}=N_{\text{laser}}\left(\;1-10^{-A}\right)$$

where $N_{\text{laser}}$ is the number of photons in the photoexcitation laser pulse. From Eq. 6, it is evident that, when the laser beam is collinear with the X‐ray beam, higher sample absorbance will result in a higher number of absorbed photons in the X‐ray probed volume, and thus in a higher SNR. The optimal absorbance is thus, in practice, determined by a compromise between increasing the SNR and minimizing other detrimental effects, such as the local increase of the sample temperature (see the Temperature jump section, below). Moreover, photoexcitation conditions should be mild enough to ensure, for example, the validity of Lambert–Beer's law:

(7) A=ϵCd

where ϵ is the molar extinction coefficient of the chromophore at the photoexcitation wavelength, C is its molar concentration, and d is the capillary diameter.

In the above, we have not discussed the contribution of refraction of the laser beam by the glass capillary. Because of refraction, the capillary focuses the laser beam in the plane orthogonal to its axis. While we will take this effect explicitly into account in the case of orthogonal geometry between the X‐ray and laser beams (see next section), in the case of collinear geometry this focusing effect is less important, provided that the X‐ray beam size is much smaller than that of the laser beam (see Fig. S1).

#### Orthogonal geometry

**Fig. 3:**
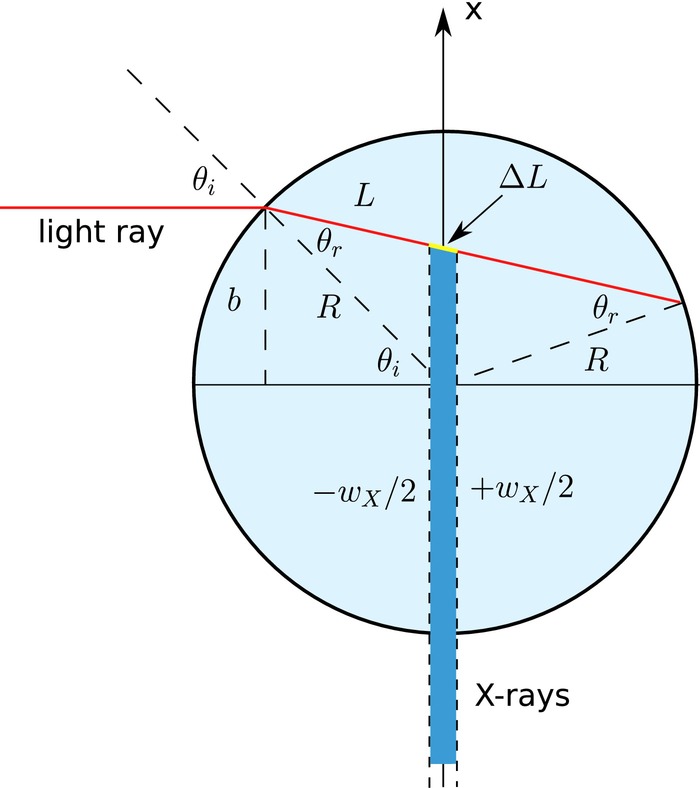
Laser path inside a round capillary in orthogonal geometry (top view). The path of a laser ray incident on the capillary with an impact parameter b is depicted in red. The capillary is filled with a liquid sample (light blue). The path ΔL of the laser ray inside the X‐ray probed volume is highlighted in yellow. The X‐ray beam is shown in dark blue.

In the case of orthogonal geometry, it is important to take into account the focusing effect of the capillary on the laser beam, as this leads to a reduction of the photoexcited volume probed by the X‐ray beam. The pathlength of laser light rays into a round capillary can be obtained by applying Snell's law and geometry (Fig. 3). The number of absorbed photons can be then estimated as follows:

(8) $$N_{\mathrm{abs}}\;=\;N_{\text{laser}}\;\;\int_{-R}^{+R}\;P\left(b\right)\;10^{-\epsilon \;C\;\left(\;L-\Delta L/2\;\right)}\;\left(1\;-\;10^{-\epsilon \;C\;\Delta L}\right)\;\;\mathrm{db}$$

where $P\left(b\right)$ is the laser spatial profile, b is the impact parameter of a given light ray, L is the pathlength of a light ray from the capillary surface to the X‐ray probed volume, and ΔL is the projection of the X‐ray beam width along the light ray path. If the X‐ray beam passes through the center of the capillary, L is given by:

(9) $$L=\frac{\;\sqrt{\;R^{2}-b^{2}\;}\;}{\cos\left(\theta_{i}-\theta_{r}\right)}$$

where R is the capillary radius, b is the impact parameter, $\theta_{i}$ is the incidence angle, and $\theta_{r}$ is the refraction angle. While ΔL is:

(10) $$\Delta L=\frac{w_{X}}{\cos\left(\theta_{i}-\theta_{r}\right)}$$

with $w_{X}$ being the X‐ray beam width. The term $P\left(b\right)$ in Eq. 8 describes how the intensity of the laser beam changes along one dimension, ie along the X‐ray propagation direction, and has the units of inverse length. The integral of $P\left(b\right)$ vs. b between −∞ and +∞ is equal to 1.

**Fig. 4:**
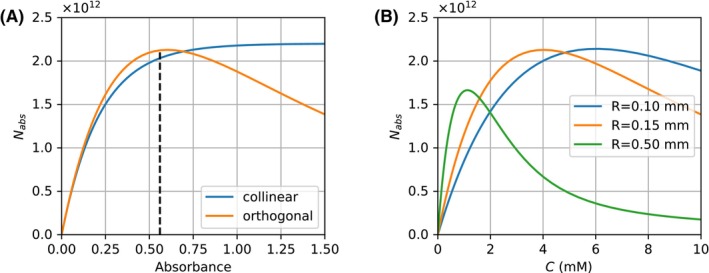
Number of absorbed photons ($N_{\mathrm{abs}}$) in the X‐ray probed volume. (A) $N_{\mathrm{abs}}$ for a liquid sample contained in a round capillary, both in the case of collinear geometry between the laser and the X‐ray beam (blue curve) and in the case of orthogonal geometry between the two beams (orange curve). $N_{\mathrm{abs}}$ is plotted as a function of the absorbance in the middle of the capillary for a capillary radius R=150μm. The vertical dashed line corresponds to the optimal absorbance (≈ 0.56 OD). (B) $N_{\mathrm{abs}}$ as a function of solute concentration in the case of orthogonal geometry for three different capillary radii: 100μm (blue), 150μm (orange), and 500μm (green). In all cases, it was assumed that the X‐ray beam intercepts the capillary axis. The values of the other parameters used in the calculation are: $N_{\text{laser}}$ = $10^{15}\;\mathrm{ph}/\text{pulse}$, ϵ = $10^{4}\;M^{-1}\cdot cm^{-1}$, $w_{X}$ = 25μm, and index of refraction n = 1.33 (aqueous solution). The laser beam profile was assumed to be Gaussian with FWHM (full width at half maximum) of 250μm along the capillary axis and 1 mm along the X‐ray propagation direction. Note that, in contrast to Eqs. 6 and 8, the calculation performed to obtain the curves reported here takes into account the finite size of the X‐ray beam also along the capillary axis.

Figure 4 reports the dependence of $N_{\mathrm{abs}}$ on chromophore concentration both in collinear geometry and in orthogonal geometry assuming a Gaussian laser beam profile. The figure shows that, while in collinear geometry $N_{\mathrm{abs}}$ increases exponentially with concentration and reaches asymptotically the number of photons in the laser pulse hitting the sample, in orthogonal geometry an optimal concentration value exists corresponding to an absorbance at the capillary center of ≈0.56.

Another way of looking at this result is in terms of the laser penetration depth, ie, the pathlength at which the light intensity has dropped by a factor 1/e due to absorption:

(11) $$d_{0}=\frac{1}{\ln\left(10\right)\;\epsilon \;C}\approx \frac{0.434}{\epsilon \;C}$$

For a concentration C=1mm and an extinction coefficient ϵ = $10\;000\;M^{-1}\cdot cm^{-1}$, the penetration depth is ≈434μm. In collinear geometry, the SNR increases as the laser penetration $d_{0}$ is reduced, while in orthogonal geometry the optimal penetration depth is $d_{0}\;\approx \;0.78\;R$ (ϵC=0.56/R). So, for a capillary radius of 0.5 mm, the optimal penetration depth is ≈390μm.

It is relevant to note that, in the above discussion, we have implicitly assumed that a collimated laser beam interacts with the sample. In practice, the laser is often focused at the capillary or close to it. In the case of orthogonal geometry, we typically use cylindrical lenses to focus the laser spot close to the sample position: a shorter focal length lens is used to focus the laser beam along the vertical direction (a focal length of 250 mm is typically used at ID09 for this purpose), while a longer focal length (typically 500 mm) lens is used to reduce the laser size along the horizontal direction. In the case of collinear geometry, a single spherical lens can be used (focal length in the 500–750 mm range). Thus, in most cases, the laser beam divergence used in experiments at ID09 is quite small (of the order of 10 mrad).

### Photoexcitation parameters

Once the sample concentration, capillary diameter and photoexcitation geometry have been chosen, the photoexcitation laser should be prepared to optimize the data collection and avoid undesired effects. As an example, it is important to properly estimate the beam size, fluence and peak power density to evaluate if multiphoton absorption could play a role. Moreover, these quantities allow to estimate the number of absorbed photons per chromophore and induced sample temperature jump as shown in the following sections.

The laser beam profile can be often approximated by a Gaussian which, in 1 dimension, is given by:

(12) $$P\left(x\right)=\frac{1}{\;\sigma \;\sqrt{2\pi }\;}\;\exp\left(-\frac{x^{2}}{2\sigma^{2}}\right)$$

where σ is the Gaussian standard deviation or root‐mean‐square (RMS) size. The laser full width at half maximum (FWHM) is equal to:

(13) $$\text{FWHM}=2\;\sqrt{2\;\ln2\;}\;\sigma \;\approx \;2.355\;\sigma$$

and the laser radius, which is the distance from the beam axis where the optical intensity drops to $1/e^{2}\;\approx \;13.5\%$, in the case of a Gaussian is equal to:

(14) $$w_{0}=2\;\sigma =\frac{\text{FWHM}}{\sqrt{2\;\ln2\;}}\;\approx \;\frac{\text{FWHM}}{1.177}$$

The laser cross‐section area is:

(15) $$A_{\text{laser}}=\frac{\pi \;w_{0}^{2}}{2}$$

The laser fluence is equal to:

(16) $$F_{\text{laser}}=\frac{E_{\text{pulse}}}{A_{\text{laser}}}=\frac{2\;E_{\text{pulse}}}{\pi \;w_{0}^{2}}=\frac{4\;\ln2}{\pi }\frac{E_{\text{pulse}}}{\mathrm{FWH}M^{2}}\;\approx \;0.88\frac{E_{\text{pulse}}}{\mathrm{FWH}M^{2}}$$

where $E_{\text{pulse}}$ is the laser energy per pulse.

In cases where the laser has an elliptical Gaussian beam profile with RMS size along x and y equal to $\sigma_{x}$ and $\sigma_{y}$, respectively, the fluence can be calculated with Eq. 16 taking into account that the beam radius is equal to:

(17) $$w_{0}=2\;\sqrt{\sigma_{x}\sigma_{y}}$$

or, equivalently, that the area is:

(18) $$A_{\text{laser}}=2\;\pi \;\sigma_{x}\;\sigma_{y}$$

The laser peak power is:

(19) $$P_{\text{peak}}=2\;\sqrt{\frac{\ln2}{\pi }}\;\frac{E_{\text{pulse}}}{\tau_{\text{pulse}}}\;\approx \;0.94\;\frac{E_{\text{pulse}}}{\tau_{\text{pulse}}}$$

where $\tau_{\text{pulse}}$ is the laser FWHM pulse duration. Finally, the laser peak power density is:

(20) $$\frac{P_{\text{peak}}}{A_{\text{laser}}}=2\;\sqrt{\frac{\ln2}{\pi }}\;\frac{F_{\text{laser}}}{\tau_{\text{pulse}}}\;\approx \;0.94\;\frac{F_{\text{laser}}}{\tau_{\text{pulse}}}$$

As mentioned in the introduction, the peak power density is an important parameter in any time‐resolved experiment. In order to minimize any multiphoton absorption effect, it is usually safe to work at peak power densities below 10 GW·cm^−2^ [4].

A critical step in a TR‐XSS experiment is thus the measurement of the laser spatial profile at sample position. This can be typically done using a beam profiler or with knife edge scans, from which the Gaussian FWHM in the horizontal and vertical direction can be estimated. If a beam profiler is used, it is important to attenuate the laser intensity sufficiently to avoid camera saturation, as this would lead to an overestimation of the beam size. In the case of knife edge scans, the laser fluence should be kept low enough to avoid damage to the knife edge itself, which can occur especially with tightly focused beams. The average laser pulse energy can be measured with a pulse energy meter positioned relatively close to sample position (after any optical element that attenuates the laser energy).

As a rule of thumb, TR‐XSS experiments on biological samples at the ID09 beamline are typically performed with fluences up to 1mJ·mm^−2^ and peak powers up to 0.02 GW·cm^−2^ using a 5 ns FWHM laser pulse duration.

### Number of photons per molecule

When choosing laser excitation condition, a trade‐off has to be found. While the TR‐XSS signal‐to‐noise is proportional to the total number of absorbed photons in the X‐ray probed volume (see Eqs. 3 and 5), high laser peak power densities can lead to artifacts. A useful parameter to keep under control when tuning the laser excitation is the number of absorbed photons per chromophore molecule $N_{\mathrm{abs}}^{\text{molecule}}$

(21) $$N_{\mathrm{abs}}^{\text{molecule}}=\frac{\text{number of absorbed photons}\;\mathrm{per}\;\text{unit area}}{\text{number of chromophore molecules}\;\mathrm{per}\;\text{unit area}}$$

which can be expressed as follows:

(22) $$N_{\mathrm{abs}}^{\text{molecule}}\;=\;\frac{\left(\frac{F_{\text{laser}}}{\mathrm{h\nu}}\right)\;\left(1\;-\;10^{-\epsilon \;C\;d}\;\right)}{n\;d}$$

where n is the number of chromophores per unit volume, h is the Planck's constant and ν is the laser photon frequency. If the chromophore concentration is expressed in moles per liter (M) and the sample thickness d is in meters, the number of chromophores per cubic meter is equal to:

(23) $$n\;\left[m^{-3}\right]=10^{3}\;N_{A}\;C\;\left[M\right]$$

where $N_{A}$ is the Avogadro number. $N_{\mathrm{abs}}^{\text{molecule}}$ can thus be rewritten as follows:

(24) $$N_{\mathrm{abs}}^{\text{molecule}}\;=\;\frac{F_{\text{laser}}\;\left[J\;m^{-2}\right]}{h\;\left[J\;s\right]\;\;\nu \;\left[s^{-1}\right]}\;\frac{1\;-\;10^{-\epsilon \;C\;d}}{\;10^{3}\;\;N_{A}\;\;C\;\left[M\right]\;\;d\;\left[m\right]}$$

For a 500 nm laser with a fluence of 1 mJ·mm^−2^ incident on a 300μm sample having 1 mm concentration and an extinction coefficient of 10 mm ^−1^·cm^−1^ (50% transmission), the average number of incident photons per chromophore molecules is ≈14 and the average number of absorbed photons per chromophore molecule is ≈7. Note than, in this example, although more than one photon is absorbed on average by a chromophore molecule, the laser peak power density is only 0.02 $\mathrm{GW}\cdot cm^{-2}$ in the case of 5 ns long laser pulses. A significantly higher value of 2 $\mathrm{GW}\cdot cm^{-2}$ would be obtained with 50 fs long laser pulses. In both cases, we are well below the typical threshold of multiphoton absorption [4], which means that, on average, a chromophore molecule will undergo seven consecutive single‐photon absorption processes within the laser pulse duration. It is not possible to know *a priori* whether this number is too large, as the threshold depends on the specific system under investigation. A good practice is to compare the results of time‐resolved experiments (TR‐XSS in particular) at different laser fluence values. This allows to experimentally determine the range of fluences that guarantee a linear dependence of the observed time‐resolved signal on the laser fluence. This is common practice at ID09 since the TR‐XSS method was developed [15]. While for many systems a linear regime is observed up to $\approx 1\;\mathrm{mJ}\cdot mm^{-2}$ and a few absorbed photons per molecule, this is by no means a general result and should be tested for each new investigated system. As a rule of thumb, it makes sense to compare TR‐XSS patterns collected in conditions where the number of absorbed photons per molecule ranges from slightly below 1 (ie, 0.5 or 0.3) up to 10 or 20. The amplitude of the TR‐XSS signal associated with the main protein conformational change at a given time‐delay is expected to increase linearly up to a certain level and then to asymptotically saturate as the fluence is further increased. A safe approach is to select the highest fluence value within the linear response interval to collect the full TR‐XSS dataset.

In cases where the photoexcitation yield is low, increasing the laser duration (rather than the energy per pulse) can be an effective technique to increase the number of absorbed photons per molecule, and thus the number of protein molecules undergoing a given conformational transition. This was successfully employed in the first TR‐XSS investigations of human hemoglobin R‐T transition [15, 16]. A 230ns long pulse was used to increase the fraction of hemoglobin molecules undergoing the quaternary structure transition and it was later shown that the estimated R‐T transition rate is identical to that obtained with a shorter 5 ns laser pulse [17] (in both cases, the laser excitation peak power density was several orders of magnitude below multiphoton absorption conditions).

There are cases in which using several consecutive photoexcitation pulses rather than a single long one is needed in order to increase the number of photoexcited proteins undergoing a structural transition. This is the case of the orange carotenoid protein (OCP), a protein whose transition from a dark inactive state to an active red state is limited by the very low photoexcitation yield of its carotenoid chromophore (0.2%) [18]. Only when the system has the time to populate a transient intermediate state, the absorption of a new photon triggers the conversion to the active red state and the large associated relative motion of the N‐terminal domain with respect to the C‐terminal one. By using a train of nanosecond long pulses with a repetition rate of 1 kHz, Andreeva *et al*. [19] have shown that it is possible to increase the number of OCP molecules undergoing this large‐scale structural transition, thus maximizing the amplitude of the TR‐XSS signal.

### Sample flowing

**Fig. 5:**
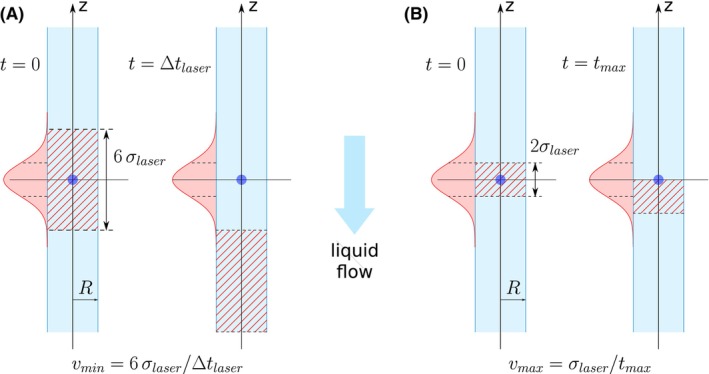
Sample flow speed regulation in a TR‐XSS (time‐resolved X‐ray solution scattering) experiment. (A) The minimum flow speed is determined by the laser size along the flow direction ($\sigma_{\text{laser}}$) and the laser repetition rate ($1/\Delta t_{\text{laser}}$). (B) The maximum flow speed depends on $\sigma_{\text{laser}}$ and on the maximum time‐delay to be explored ($t_{\max}$). The laser spatial profile (red) is represented as a Gaussian with RMS (root‐mean‐square) size $\sigma_{\text{laser}}$. The X‐ray spot size (dark blue circle) intercepts the capillary in its axis. In panel A, the whole photoexcited area (height $\approx 6\;\sigma_{\text{laser}}$) is highlighted with red lines. In panel B, the highlighted area corresponds to the area (height $\approx 2\;\sigma_{\text{laser}}$) that receives the central part of the laser pulse.

In order to avoid accumulation of radiation damage effects in TR‐XSS experiments, protein solutions are typically flown through capillaries by means of peristaltic or syringe pumps. The sample flow speed has to be high enough to ensure complete sample refresh in between consecutive laser pulses and low enough to ensure access to the desired pump‐probe time‐delays (Fig. 5). Quantitatively, the flow speed has to be higher than:

(25) $$v_{\min}=\frac{\;\alpha \;\sigma_{\text{laser}}\;}{\Delta t_{\text{laser}}}$$

and lower than:

(26) $$v_{\max}=\frac{\;\beta \;\sigma_{\text{laser}}\;}{t_{\max}}$$

where $\sigma_{\text{laser}}$ is the laser RMS width in the direction of the sample flow, $\Delta t_{\text{laser}}$ is the time between consecutive laser pulses (inverse of the repetition rate) and $t_{\max}$ is the longest time‐delay to be investigated. α and β are proportionality constants. Typical values used by several investigators are α between 4 and 6 and β between 1 and 2. Conservative values are α = 6 and β = 1. Note that in order to have $v_{\min}<v_{\max}$, the above equations imply that $t_{\max}<\beta /\alpha \;\Delta t_{\text{laser}}\approx 1/6\;\Delta t_{\text{laser}}$.

The above equations do not, however, take into account that, in the case of laminar flow, the fluid velocity increases toward the center of the tube. The fluid velocity profile is given by:

(27) $$v\left(r\right)=v_{0}\left(1-\frac{r^{2}}{R^{2}}\right)$$

where r is the radial distance from the capillary axis and $v_{0}$ is the on‐axis flow speed. The average flow speed along the tube is:

(28) $$v_{\mathrm{ave}}=\frac{v_{0}}{\;2}$$

Eq. 27 implies that the flow speed at the capillary wall is zero, which can lead to undesired effects, such as protein aggregation at the capillary surface due to multiple excitations of protein molecules close to the capillary wall. Aggregation typically occurs at the laser incidence surface, where the sample temperature increase is most significant (see the Temperature jump section, below). Using an orthogonal geometry is clearly beneficial from this point of view, as the X‐ray beam will not probe the sample in that region. Co‐flow approaches can also be used to minimize these effects when dealing with particularly sensitive samples [20]. Integrating Eq. 27 over the capillary cross section, the following relationship between the sample flow rate Q and the average flow speed $v_{\mathrm{ave}}$ is obtained:

(29) $$Q=\pi \;R^{2}\;\left(\frac{v_{0}}{\;2}\right)$$

If we take a typical case in which R=150μm and $Q<0.3\;\mathrm{ml}\cdot \min^{-1}$, we obtain from the above equation that the average flow speed is less than 0.3 m·s^−1^, which corresponds to a Reynolds number of 100. This confirms that the sample flow is laminar, as turbulences are typically observed at a Reynolds number greater than 2000 [21].

### Temperature jump

The absorption of a laser pulse by a chromophore eventually leads to the release of heat into the solvent on ps–ns timescales. This produces a temperature jump (T‐jump) that contributes to the time‐resolved X‐ray scattering signal and can potentially perturb the protein structure or the reaction kinetics of interest. In general, only a fraction of the absorbed photon energy ends up in the solvent. This fraction $\phi_{\text{heat}}$ is determined by the different microscopic processes (photochemical modifications, internal conversion, vibrational relaxation, fluorescence, phosphorescence, etc.) occurring in a given chromophore after photon absorption. The relation between the absorbed energy per unit volume and the temperature jump ΔT is thus given by:

(30) $$\phi_{\text{heat}}\;\frac{dE_{\mathrm{abs}}}{\mathrm{dV}}=\rho \;c_{P}\;\Delta T$$

where ρ is the solvent density and $c_{P}$ is the solvent‐specific heat capacity at constant pressure. For a laser pulse with energy $E_{\text{laser}}$ and area $A_{\text{laser}}$ propagating along y, making use of the Lambert–Beer's law, we can write the energy absorbed per unit volume as follows:

(31) $$\frac{dE_{\mathrm{abs}}}{\mathrm{dV}}=-\frac{1}{A_{\text{laser}}}\;\frac{dE_{\text{laser}}}{\mathrm{dy}}=\alpha \left(\lambda \right)\;\frac{E_{\text{laser}}}{A_{\text{laser}}}$$

where $\alpha \left(\lambda \right)$ is the chromophore absorption coefficient (inverse of the penetration depth) at the laser wavelength (λ). Combining Eqs. 30 and 31 we have:

(32) $$\Delta T\;=\;\frac{\phi_{\text{heat}}\;\;\alpha \left(\lambda \right)\;E_{\text{laser}}}{\rho \;\;c_{P}\;A_{\text{laser}}}$$

The laser pulse energy and thus the T‐jump decay exponentially as the laser penetrates into the capillary as follows:

(33) $$\Delta T\left(d\right)=\Delta T\left(0\right)\;e^{-\alpha \left(\lambda \right)\;d}$$

For water $\rho \approx 10^{3}\;\mathrm{kg}\cdot m^{-3}$ and $c_{p}\;\approx \;4.2\times 10^{3}\;J\cdot kg^{-1}\cdot K^{-1}$. In the case of direct solvent excitation using near‐infrared (NIR) light at a fluence $F_{\text{laser}}=1\;\mathrm{mJ}\cdot mm^{-2}$ and $\alpha =10\;cm^{-1}$ (weak NIR absorption), the temperature jump at the capillary surface is:

(34) $$\Delta T\left(0\right)\;\approx \;\frac{10^{3}\times 10^{3}}{4.2\times 10^{6}}\;\approx \;0.24\;K$$

After heat deposition, the thermal profile evolves by diffusion with a timescale:

(35) $$\tau_{\text{heat}}∼\frac{w_{X}^{2}}{D_{\text{heat}}}$$

where $w_{X}$ is the X‐ray spot size and $D_{\text{heat}}\;\approx \;1.4\times 10^{-7}\;m^{2}\cdot s^{-1}$ is the heat diffusion coefficient in water. For $w_{X}=25\;\mathrm{\mu m}$, the diffusion time $\tau_{\text{diff}}$ is on the order of a few milliseconds, meaning that the T‐jump is effectively static over typical μs pump–probe delays but relaxes between shots if the repetition period is longer or if fresh sample is flowed in. Repetitive heating may accumulate if the flow speed is not high enough. Moreover, ΔT≳5−10K may induce viscosity changes, bubble formation, or partial protein denaturation. Because of that, in most cases, it is desirable to tune the laser or modify the sample absorbance in order to keep the T‐jump per pulse below ~1 degree, thus minimizing thermal artifacts while preserving sufficient photoexcitation for time‐resolved structural measurements. It is also important to verify that the flow speed is high enough to avoid repetitive heating in the sample volume. Finally, avoiding large laser beam divergences is beneficial for minimizing significant temperature gradients within the sample.

The fraction $\phi_{\text{heat}}$ in Eq. 30, which determines the amount of excess energy deposited by the laser into the solvent, can be experimentally determined by comparing the TR‐XSS signal of the sample under study with either static XSS measurements performed at different well‐controlled temperatures and/or by TR‐XSS measurements in which an infrared laser is used to deposit energy directly into the solvent molecules. By comparing the change in the solvent scattering peak region (which is at ≈2 Å ^−1^ in the case of water) associated with a well‐known solvent temperature change ΔT with the energy absorbed from the laser energy, one can obtain $\phi_{\text{heat}}$ from Eq. 30.

## Tips & tricks

- The laser size should be measured at the sample position in order to estimate the fluence, peak power density, number of absorbed photons per molecule, or the solvent temperature jump. This can be done using a beam profiler or by performing knife‐edge scans. If a beam profiler is used, it is important to attenuate the laser intensity enough to avoid any camera saturation. Failing to do that will result in an overestimation of the beam size. In the case of knife‐edge scans, the laser fluence used for the scans should be low enough to avoid any significant damage on the knife edge itself, which can happen, especially when working with tightly focused beams.
- In the case of orthogonal photoexcitation geometry, it might be useful to offset the X‐rays with respect to the capillary axis. Indeed, if the laser penetration depth is smaller than the capillary radius, probing the sample with X‐rays closer to the capillary surface hit by the laser, might lead to an increase in the SNR. A compromise has to be found, since the X‐ray pathlength through the capillary gets smaller as the capillary vs. X‐ray offset is increased, thus leading to a reduction of the sample scattered intensity with respect to the background scattering.
- Sample flowing is important to avoid artifacts in the data. If a peristaltic pump is used, the average flow rate vs. the rotor speed should be measured in advance using the same circuit that will be employed in the TR‐XSS experiment.
- An excessive temperature jump at the capillary surface should be avoided in order to prevent protein denaturation and aggregation. Either the fluence or the concentration of the laser absorbing chromophore (or both) should be reduced if the temperature jump is too high for the specific system or reaction investigated.
- Having an estimate of the system photoexcitation yield is very important to properly evaluate the feasibility of a TR‐XSS experiment. Performing a time‐resolved optical absorption measurement using a pump laser similar to the one that will be used in the TR‐XSS experiment is recommended.

## Proof‐of‐principle TR‐XSS dataset

In order to show the usefulness of the methods detailed above, we consider here the case of a biological system recently investigated at ID09 with TR‐XSS [12]. As already mentioned, *Thermus thermophilus* CarH (*Tt*CarH) is a protein photoreceptor containing the AdoCbl chromophore, which is a vitamin $B_{12}$ derivative. In the absence of light, CarH is a tetramer bound to DNA, while under sun exposure, photon absorption by AdoCbl triggers a series of ultrafast structural rearrangements at the level of the chromophore that propagate into the tertiary and quaternary protein structure and ultimately lead to the protein tetramer‐to‐monomer transition and detachment from DNA [12] in the millisecond‐to‐second timescale. This molecular machinery is used by *Thermus thermophilus* to control the expression of carotenoid by direct CarH interaction with the carotenoid expression gene.

**Fig. 6:**
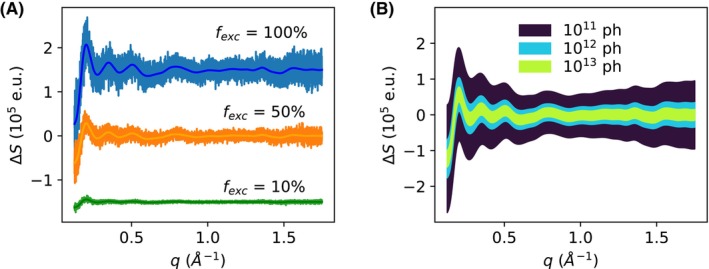
Calculated CarH light–dark X‐ray solution difference pattern. (A) Comparison of the calculated TR‐XSS (time‐resolved X‐ray solution scattering) signal at three different photoexcitation yield ($\kappa_{\text{light}}$) values (1, 0.5, and 0.1). (B) Effect of the total number of incident photons on the SNR (signal‐to‐noise ratio). The noise on the calculated signal is based on the assumption of shot‐noise‐limited data as described in the Supporting Information. The TR‐XSS signal was calculated using the PDB (Protein Data Bank) structures *8c73* (dark state) and *8c76* (light‐activated state) [22] and assuming a protein concentration of 240 μm, a capillary diameter of 1 mm, and typical parameters for the X‐ray detectors (pixel size 88 μm, sample‐to‐detector distance 350 mm and X‐ray photon energy of 14.7 keV). In panel B, the noise on the difference pattern has been smoothed via a cubic spline and corresponds to the line thickness of each curve.

As it is often the case when conducting a new TR‐XSS experiment, models of the protein structure in its dark state and in one of the light‐activated states were already known in advance [22]. This prior knowledge allows estimating the expected TR‐XSS signal, since the solution scattering pattern can be easily estimated from an available PDB file through publicly available software like CRYSOL3 [23]. Using the theory developed in the Supplementary Text, it is possible to evaluate the noise on such calculated signal (see Eq. 4). Figure 6 reports the result of the calculation obtained using the *Tt*CarH PDB structures *8c73* and *8c76* [22]. The amplitude of the TR‐XSS signal is proportional to the photoexcitation yield $\kappa_{\text{light}}$ and to illustrate this effect, Fig. 6A reports the expected CarH signal corresponding to three different photoexcitation yields (1, 0.5, and 0.1). The SNR depends on the total number of photons incident on the sample that are then scattered and accumulated in each detector image. Figure 6B shows how the SNR varies as the number of incident photons is changed between $10^{11}$ to $10^{13}$. It is evident that $10^{13}$ photons are needed in order to obtain a high enough SNR to resolve the characteristic oscillations of the CarH TR‐XSS signal. In the case of 10μs long X‐ray pulses ($\approx 2\times 10^{11}$ photons/pulse) used for the data collection on CarH, this can be achieved by accumulating the scattering signal from 50 pulses on the detector, which at the 2 Hz employed repetition rate corresponds to an exposure time per image of 25 s. The SNR can be further improved by averaging the signal over different collected images. However, in order to improve the scattering signal by a factor of 5, one needs to collect both the scattering after photoexcitation ($S_{\text{light}}$) and the reference ($S_{\text{dark}}$) 25 times. It is clear that, depending on the investigated system, the particular considered structural change and the available experimental conditions, this kind of calculation can allow estimating the feasibility of a TR‐XSS experiment.

**Fig. 7:**
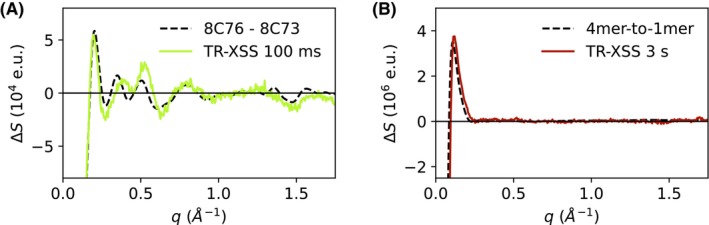
Comparison of experimental and calculated TR‐XSS (time‐resolved X‐ray solution scattering) signals for *Tt*CarH (*Thermus thermophilus* CarH). (A) Experimental TR‐XSS signal measured at 100 ms from photoexcitation (green line) and signal calculated from PDB (Protein Data Bank) models of the light and dark state (dashed line). (B) Experimental TR‐XSS signal at 3 s (red line) and calculated signal corresponding to the CarH tetramer‐to‐monomer transition (dashed line).

A comparison between the calculated signal obtained from the PDB structures *8c73* and *8c76* with the experimentally determined TR‐XSS signal at 100 ms from photoexcitation [12] is reported in Fig. 7A. Although the light‐activated state PDB structure is not representative of the CarH structure at 100 ms, the calculated and experimental signals are in reasonable overall agreement. An analogous comparison is reported in Fig. 7B, where the experimental signal measured at 3 s from photoexcitation is compared with the calculated difference corresponding to the tetramer‐to‐monomer transition of CarH. Note that, the amplitude of the TR‐XSS signal (reported in Fig. 7B in electron units per CarH tetramer) is much larger than that corresponding to the tertiary structural change at 100 ms.

The 100 ms TR‐XSS dataset was obtained using a laser pulse duration of 5 ns, a laser wavelength of 532 nm, and a laser fluence of 0.5 $\mathrm{mJ}\cdot mm^{-2}$ (peak power density 0.009 $\mathrm{GW}\cdot cm^{-2}$). The sample was flowing through a 1.5 mm capillary at a flow rate of 80 μl·min^−1^ and the sample was exposed to X‐ray vs. laser pump‐probe pairs in orthogonal geometry at a repetition rate of 2 Hz. According to the equations reported in the text, the flow rate was high enough to ensure the refresh of the sample every 500 ms and low enough to avoid significant displacement of the photoexcited volume after 100 ms from photoexcitation. The sample absorbance in the middle of the capillary was 0.14, limited in this case by the max available protein concentration. In the above conditions, there were ~3.5 absorbed photons per chromophore molecule according to Eq. 24. Finally, in view of the low sample absorbance at the excitation wavelength, the temperature jump was well below 1 degree even at the capillary surface.

## Conflict of interest

The author declares no conflict of interest.

## Author contributions

ML conceived and designed the study, performed the experiments, analyzed the data, and wrote the article.

## Supporting information


**Fig. S1.** Laser path inside a round capillary in collinear geometry (top view). The path of laser rays incident on the capillary are depicted in red. The capillary is filled with a liquid sample (light blue). The X‐ray beam (X‐ray solution scattering beam) is shown in dark blue. If the laser beam is collimated and the X‐ray beam size is much smaller than the laser size, the refraction of the laser light by the sample leads to a minor increase of the fluence in the back part of the capillary.

## Data Availability

The TR‐XSS datasets analyzed during the current study are publicly available in the ESRF Data Portal with DOI 10.15151/ESRF‐ES‐704502367 and 10.15151/ESRF‐ES‐1437867878. Additional details on sample preparation and experimental conditions can be found in Rios‐Santacruz *et al*. [12].
