# Supplementary material for: Optimizing photoexcitation conditions for time‐resolved X‐ray solution scattering experiments
Source: FEBS Open Bio. 2026 Jul 15:10.1002/2211-5463.70304. Online ahead of print. doi: 10.1002/2211-5463.70304 (PMC13399038; doi:10.1002/2211-5463.70304)
Supplement: Supplementary file 1 — Fig. S1. Laser path inside a round capillary in collinear geometry (top view). The path of laser rays incident on the capillary are depicted in red. The capillary is filled with a liquid sample (light blue). The X‐ray beam (X‐ray solution scattering beam) is shown in dark blue. If the laser beam is collimated and the X‐ray beam size is much smaller than the laser size, the refraction of the laser light by the sample leads to a minor increase of the fluence in the back part of the capillary. [file FEB4-9999-0-s001.pdf]

Supplementary Information

Optimizing photoexcitation conditions for time-resolved X-ray solution scattering experiments

Matteo Levantino[1]

[1]ESRF – The European Synchrotron, 71 avenue des Martyrs, CS 40220, F-38043 Grenoble, France.

Supplementary Text

Scattering intensity

If an X-ray beam of intensity I_0 photons per second is incident on a sample which is large enough to intercept the whole beam, then the number of photons scattered per second on a detector pixel is given by:

$$I_{pixel} \;=\; I_0 \; c \; d \; \Delta\Omega_{pixel} \left(\frac{d\sigma}{d\Omega}\right) \tag{S1}$$

where c is the number of molecules per unit volume in the sample, d is the sample thickness, $\Delta\Omega_{pixel}$ is the solid angle subtended by the pixel and $(d\sigma/d\Omega)$ is the sample differential cross-section [1]. The scattered intensity is often rewritten in terms of the dimensionless quantity $S(\theta)$ as:

$$I_{pixel} \;=\; I_0 \; c \; d \; \Delta\Omega_{pixel} \; r_e^2 \; S(\theta) \tag{S2}$$

where r_e^2 is the classical electron radius.

The projection of the pixel area is equal to the solid angle $\Delta\Omega_{pixel}$ scaled by the square of the sample-to-pixel distance R:

$$\Delta A_{pixel} \; cos\,\theta \;=\; R^2 \; \Delta\Omega_{pixel} \tag{S3}$$

Introducing the sample-to-detector perpendicular distance $D = R\; cos\,\theta$, we have that:

$$\Delta\Omega_{pixel} \;=\; \Delta A_{pixel} \left(\frac{cos^3\,\theta}{D^2}\right) \tag{S4}$$

Substituting Eq. S4 into Eq. S2 we obtain an expression which depends only on $S(\theta)$ and other easily accessible experimental parameters:

$$I_{pixel} \;=\; I_0 \; c \; d \; \Delta A_{pixel} \; \frac{cos^3\theta}{D^2} \; r_e^2 \; S(\theta) \tag{S5}$$

The number of photons scattered in an annular ring of radius $r(\theta)$ and thickness Δr is the product of I_{pixel} times the number of pixels in the annular ring, which for Δr greater than or equal to the pixel size Δl_{pixel}, can be approximated by $2\,\pi\, r(\theta)\,\Delta r$, thus:

$$I(\theta) \;=\; I_0\; c\; d\; \Delta A_{pixel}\; \frac{cos^3\theta}{D^2}\; r_e^2\; S(\theta)\; 2\pi\, r(\theta)\, \Delta r \tag{S6}$$

where $r(\theta) = D\, tan\,\theta$. We will consider here for simplicity the case of $\Delta r \approx \Delta l_{pixel}$, thus:

$$I(\theta) \;=\; I_0\; c\; d\; \Delta A_{pixel}\; \frac{cos^3\theta}{D^2}\; r_e^2\; S(\theta)\; 2\,\pi\, D\; \frac{sin\,\theta}{cos\,\theta}\; \Delta l_{pixel} \tag{S7}$$

Rearranging the terms, we finally obtain the following expression for the number of photons scattered per unit time at an angle θ:

$$I(\theta) \;=\; 2\,\pi\, r_e^2\; \frac{I_0\; c\; d\; \Delta l_{pixel}^3}{D}\; sin\,\theta\; cos^2\theta\; S(\theta) \tag{S8}$$

This expression is reported here as a function of the scattering angle θ, but can of course equivalently be expressed in terms of the scattering vector magnitude q:

$$q \;=\; \frac{4\,\pi}{\lambda}\, sin\left(\frac{\theta}{2}\right) \tag{S9}$$

where λ is the X-ray wavelength.

In the case of a protein solution, $S(q)$ in Eq. S8 has the following main contributions: the scattering from the solvent (buffer) molecules, the scattering from the proteins and the background scattering (air, sample holder, etc.). All these contributions can be calculated starting from atomic form factors and the total scattering $S(q)$ can be approximated as the sum of these three contributions:

$$S(q) \;=\; S_{solv}(q) \;+\; S_{prot}(q) \;+\; S_{bck}(q) \tag{S10}$$

The solvent contribution can be written as:

$$S_{solv}(q) \;=\; N_{solv}\; s_{solv}(q) \tag{S11}$$

where N_{solv} is the number of solvent molecules in the X-ray illuminated volume and $s_{solv}(q)$ is the scattering contribution per solvent molecule. On the other hand, in a TR-XSS (time-resolved X-ray solution scattering) experiment the protein contribution can be split in two parts: the scattering from proteins undergoing a structural change after photoexcitation and that from proteins that are still in the "dark" state (equilibrium state in the absence of photoexcitation). We can then write Eq. S10 as:

$$S(q) \;=\; N_{solv}\; s_{solv}(q) \;+\; N_{prot}^{light}\; s_{prot}^{light}(q) \;+\; N_{prot}^{dark}\; s_{prot}^{dark}(q) \;+\; S_{bck}(q) \tag{S12}$$

where N_{prot}^{light} and N_{prot}^{dark} are the number of protein molecules in the photoexcited and dark state, respectively, and $s_{prot}^{light}(q)$ and $s_{prot}^{dark}(q)$ are the corresponding scattering contribution per protein molecule.

Signal-to-noise ratio

In a TR-XSS (time-resolved X-ray solution scattering) experiment the signal is proportional to the difference between the scattering pattern of the sample after photoexcitation, $S_{light}(q,\, t)$, minus that before photoexcitation, $S_{dark}(q)$:

$$\Delta S(q,\, t) \;=\; S_{light}(q,\, t) \;-\; S_{dark}(q) \tag{S13}$$

where all terms in Eq. S13 are scaled with respect to the scattering of a single electron (electron units). Both $S_{light}(q,\, t)$ and $S_{dark}(q)$ can be written as the sum of contributions from solvent

molecules and protein molecules, plus a background (air scattering, capillary scattering, etc.). Following Eq. S12 reported in Supplementary Material 1, we can write $S_{light}(q,\, t)$ as:

$$S_{light}(q,\, t) \;=\; N_{solv}\; s_{solv}(q) \;+\; N_{prot}^{light}\; s_{prot}^{light}(q,t) \;+\; N_{prot}^{dark}\; s_{prot}^{dark}(q) \;+\; S_{bck}(q) \tag{S14}$$

and $S_{dark}(q)$ as:

$$S_{dark}(q) = \; N_{solv}\; s_{solv}(q) \;+\; N_{prot}\; s_{prot}^{dark}(q) \;+\; S_{bck}(q) \tag{S15}$$

where N_{prot}^{tot} is the total number of proteins in the X-ray probed volume. Note that, in the above equation, we have neglected any solute-solvent cross-term contribution and any direct or indirect effect of the laser on the solvent structure. This is an approximation that works reasonably well at q values where protein scattering is large with respect to solvent scattering. Using Eqs. S14 and S15, we can rewrite Eq. S13 as:

$$\Delta S(q,\, t) \;=\; N_{prot}^{light}\; s_{prot}^{light}(q,t) \;+\; N_{prot}^{dark}\; s_{prot}^{dark}(q) \;-\; (N_{prot}^{light} \;+\; N_{prot}^{dark})\; s_{prot}^{dark}(q) \tag{S16}$$

and finally obtain the following equation for $\Delta S(q,t)$:

$$\Delta S(q,\, t) \;=\; N_{prot}^{light}\; \left[s_{prot}^{light}(q,t) \;-\, s_{prot}^{dark}(q) \right] \tag{S17}$$

While the signal is given by Eq. S17, which finally depends only on the protein scattering, it is important to take into account all contributions to the scattering intensity in order to estimate the noise. Indeed the noise on $\Delta S(q,\, t)$ is given by:

$$\sigma_{\Delta S} \;=\; \sqrt{\sigma_{S_{light}}^2 \;+\; \sigma_{S_{dark}}^2} \tag{S18}$$

In the hypothesis of shot-noise limited measurements, the scattered intensity follows a Poisson distribution, thus:

$$\sigma_{\Delta S} \;=\; \sqrt{S_{light}(q,\, t) + \; S_{dark}(q)} \tag{S19}$$

Combining Eqs. S14, S15, S17 and S19, we obtain the following expression for the signal-to-noise ratio (SNR):

$$SNR \;=\; \frac{N_{prot}^{light}\; \left[s_{prot}^{light}(q,t) \;-\, s_{prot}^{dark}(q) \right]}{\sqrt{2\; N_{solv}\; s_{solv}(q) \;+\; N_{prot}^{light}\; s_{prot}^{light}(q,t) \;+\; \left[N_{prot}^{dark} \;+\; N_{prot} \right]\; s_{prot}^{dark}(q) \;+\; 2\; S_{bck}(q)}} \tag{S20}$$

From the above equation it is evident that, while the solvent and background do not contribute to the signal within the approximation considered here, they significantly impact the noise and SNR.

For relatively small structural changes, $s_{prot}^{light} \approx s_{prot}^{dark}$ and the SNR can be approximated as:

$$SNR \;=\; \frac{N_{prot}^{light}\; \left[s_{prot}^{light}(q,t) \;-\, s_{prot}^{dark}(q) \right]}{\sqrt{2\; [N_{solv}\, s_{solv}(q) \;+ N_{prot}\; s_{prot}(q) \;+\; S_{bck}(q)]}} \tag{S21}$$

References

[1] Als-Nielsen J. and McMorrow D. *Elements of Modern X-ray Physics.* Wiley, 2011.

Supplementary Figure 1

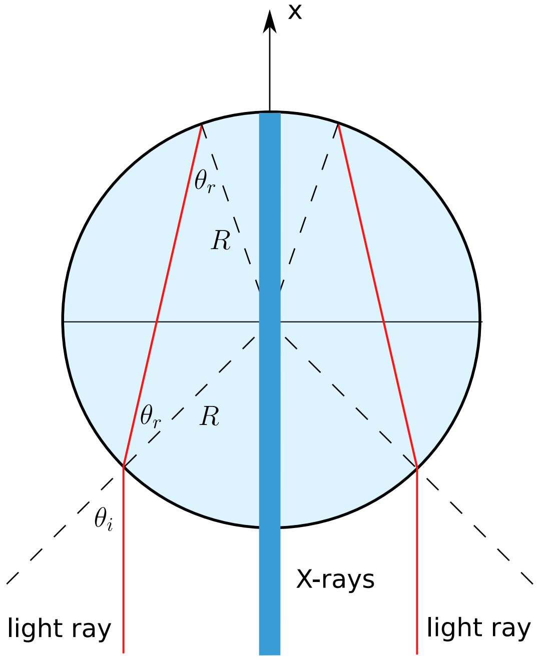

Fig. S1. Laser path inside a round capillary in collinear geometry (top view). The path of laser rays incident on the capillary are depicted in red. The capillary is filled with a liquid sample (light blue). The X-ray beam (X-ray solution scattering beam) is shown in dark blue. If the laser beam is collimated and the X-ray beam size is much smaller than the laser size, the refraction of the laser light by the sample leads to a minor increase of the fluence in the back part of the capillary.
